# 2018 Position Paper on Biosimilars of the Greek Rheumatology Society and Professional Association of Greek Rheumatologists

**DOI:** 10.31138/mjr.30.1.82

**Published:** 2019-05-31

**Authors:** Dimos Patrikos, Kyriaki Boki, Dimitrios Boumpas, Dimitrios Vassilopoulos

**Affiliations:** 1 Rheumatology Unit, Metropolitan Hospital, Piraeus, Greece; 2 Rheumatology Unit, Sismanoglio Hospital, Athens, Greece; 3 National and Kapodistrian University of Athens, School of Medicine, Attikon University Hospital, Athens, Greece; 4 Joint Rheumatology Program, Clinical Immunology-Rheumatology Unit, 2nd Department of Medicine and Laboratory, National and Kapodistrian University of Athens School of Medicine, Hippokration General Hospital, Athens, Greece

**Keywords:** Position, biosimilars, rheumatology

## INTRODUCTION

Biosimilars are biologic drugs that are similar but not identical (like generics) to the “original” or “reference” biologics, regarding their quality, safety and efficacy. The introduction of biosimilars in clinical practice offers to patients and payers the opportunity to have access to a wider range of efficacious and lower cost therapies for the management of rheumatic diseases.

In this Position paper, we present an updated statement on biosimilars based on the previous one issued by the Greek Rheumatology Society and Professional Association of Greek Rheumatologists three years ago (2015)^1^ and the most recent position papers of International Scientific Societies.^2–4^

## POSITION 1: PRESCRIPTION WITH THE BRAND NAME.

### All biologics (reference and biosimilars) must be prescribed with their brand names, and not with the International Non-proprietary Name (INN).

This statement is in accordance to the existing guidelines in order to prevent substitution of a biosimilar by the pharmacist. It is important, to record in the patient’s file the batch number in order to improve the traceability and identification of the administered biologic.

## POSITION 2: PRESCRIPTION FOR CLINICAL REASONS.

### The clinical efficacy and safety should guide the prescription of any biologic agent.

The prescription of any biologic agent must be individualized mainly on clinical grounds and not only cost savings. In general, when prescribing physicians make treatment decisions, they should always take into account the efficacy, safety and the cost-benefit ratio of the biologic.

1. New patients: The Greek Rheumatology Society supports the addition of biosimilars as an equal therapeutic choice for patients starting a new biologic therapy.
2. Interchangeability: The decision to switch a patient who is already on a reference biologic to its biosimilar must be individualized. It is important to ensure that patients who had a good response to an existing reference drug and switch to a biosimilar due to non-medical reasons (regulatory issues, cost etc.), have a close follow-up for effectiveness and safety. If they don’t achieve a good response after switching, they must have the option to return to the reference biologic. Unless adequate data are available for the safety and efficacy of multiple switches (>2) between reference and biosimilar products, these are not acceptable due to the challenges in tracing their possible adverse events and the absence of data proving their efficacy.
3. Extrapolation: Despite previous concerns, the extension of use of biosimilars in other rheumatic diseases for which the reference product is indicated, is acceptable today.

## POSITION 3: SUBSTITUTION ONLY AFTER THE PRESCRIBERS’ CONSENT.

### In case of non-availability of the prescribed biologic (reference or biosimilar) the pharmacist must first contact and obtain the prescribing physicians’ approval before temporarily dispensing an alternative biologic (reference or biosimilar).

The patient must be always informed about discussions made regarding their biologic treatment taking into consideration the possible nocebo effect. Patients should be able to check with the prescribing physician and pharmacist that the prescribed medication has been actually dispensed.

## POSITION 4: A TREATMENT DECISION IS ALWAYS A SHARED PROCESS BETWEEN PATIENTS AND THEIR RHEUMATOLOGISTS.

### Physicians must always inform their patients regarding the course of their disease and the medications that have been prescribed.

The Greek Rheumatology Society supports the shared decision-making process, during which all treatment choices should be first discussed with the patient, as well as decisions and reasons for drug switches; the switch should be made with the patient’s collaboration and consent. Prescribing physicians must keep patients aware of the need to report any potential side effects associated with the use of the prescribed biosimilar.

## POSITION 5: REGISTRATION TO A NATIONAL OR ANOTHER BIOLOGICS REGISTRY.

### It is imperative that there is close monitoring of all patients receiving biosimilars in terms of their safety and efficacy in order to protect patients and provide the long-term evidence needed to ensure the safety and efficacy of these medications in daily clinical practice.

The European Medicines Agency (EMA) suggests that all authorized biologic agent manufacturers have to participate in pharmacovigilance trials as well as in real life registries which have been planned to observe the safety of the reference products. The Greek Rheumatology Society supports the recommendation that all patients starting for the first time or switching to a biosimilar, must be registered in a National or other Registry in order to collect the same safety data that are being collected for the reference products. These data will allow patients and clinicians to make treatment decisions based on local, real life safety and efficacy data.

## OTHER ISSUES REGARDING BIOSIMILARS

Although it is recognized that prior to the market authorization of a biosimilar, a strict comparison to the reference product in terms of efficacy and safety has been made, there are some objections regarding the level of evidence. For example, bio-equivalence trials for biosimi-lars usually include a small number of patients, and there are no separate clinical trials for each indication of the reference product, which limits the power of evidence that supports the extrapolation from one indication to all other indications. Despite the reassuring results of the published studies so far, we need more long-term safety and efficacy data for patients who had previously a good response to the reference product and were then switched to a biosimilar. Furthermore, there is lack of data for the long-term safety of biosimilars, which potentially differ in their immunogenicity. Given the complexity and the high level of expertise needed for the development of such medications, the importance of a reliable biosimilar manufacturer is self-evident.

Physicians are determined to ensure quality care with the minimum cost for the patient and the society. However, prescribing is tailored to the needs of the patient and based upon clinical indications and not for cost-saving considerations. The Greek Rheumatology Society and Professional Association of Greek Rheumatologists support cost-savings efforts but cannot be involved in discussions regarding costs which are the sole responsibility of the state and the insurance agencies.
